# Effects of a periodized circuit training protocol delivered by telerehabilitation compared to face-to-face method for knee osteoarthritis: a protocol for a non-inferiority randomized controlled trial

**DOI:** 10.1186/s13063-021-05856-8

**Published:** 2021-12-06

**Authors:** Jéssica Bianca Aily, Aline Castilho de Almeida, Marcos de Noronha, Stela Marcia Mattiello

**Affiliations:** 1 Department of Physical Therapy, Federal University of São Carlos (UFSCar), São Carlos, Brazil; 2 Rural Health School, Community and Allied Health Department, La Trobe University, Melbourne, Australia

**Keywords:** Intermuscular fat, Computed tomography, Circuit training, Social isolation, Telerehabilitation, Osteoarthritis, Physical therapy, Randomized controlled trial

## Abstract

**Background:**

Regular exercise is an effective method for reducing pain and disability in patients with knee osteoarthritis (OA), as well as improving body composition. Thus, a combination of both resistance and aerobic training (circuit training) has shown to be promising for this population. However, access to physical therapy is limited by physical distance, social isolation, and/or treatment costs. Remote rehabilitation seems to be an effective way to minimize these barriers, but the benefits are dependent on the participants’ adherence to the interventions provided at a distance. The objectives of this protocol are to compare the effects of a periodized circuit training applied via telerehabilitation with the same protocol applied in the face-to-face model for individuals with knee OA.

**Methods:**

This study presents a single-blinded protocol for a non-inferiority randomized controlled trial. One hundred participants diagnosed with knee OA (grades II and III Kellgren and Lawrence system), aged 40 years or more, and BMI < 30 kg/m^2^ will be randomly divided into two groups: telerehabilitation (TR) and face-to-face (FtF) circuit training. The FtF group will perform a 14-week periodized circuit training protocol supervised by a physical therapist, 3 times a week. The TR group will perform the same exercise protocol at home, at least 3 times a week. In addition, the TR group will be able to follow the execution and orientations of the exercises by DVD, a website, and online file sharing tools, and they will receive periodic phone calls in order to motivate, clarify, and inform some aspects of knee OA. The primary outcomes are changes in self-reported pain intensity (visual analog scale (VAS)) and physical function (Western Ontario and McMaster Universities Osteoarthritis Index (WOMAC)), with a primary end-point of 14 weeks and a secondary end-point of 26 weeks. Secondary outcomes include changes in other clinical outcomes, in morphological characteristics, adherence, acceptability, and treatment perspective.

**Discussion:**

A circuit training through telerehabilitation may contribute to developing early intervention in the causative and potentiating factors of the knee OA, verifying the effects of a low-cost, non-pharmacological and non-invasive treatment.

**Trial registration:**

Brazilian Registry of Clinical Trials (ReBEC) ID: RBR-662hn2. Registered on 31 March 2019. Link: http://www.ensaiosclinicos.gov.br; Universal Trial Number (UTN) of World Health Organization: U1111-1230-9517.

**Supplementary Information:**

The online version contains supplementary material available at 10.1186/s13063-021-05856-8.

## Administrative information

Note: the numbers in curly brackets in this protocol refer to SPIRIT checklist item number. The order of the items has been modified to group similar items (see http://www.equator-network.org/reporting-guidelines/spirit-2013-statement-defining-standard-protocol-items-for-clinical-trials/).

**Table:** 

Title {1}	Effects of a periodized circuit training protocol delivered by telerehabilitation compared to face-to-face method for knee osteoarthritis: a protocol for a randomized controlled trial
Trial registration {2a and 2b}.	RBR-662hn2 – Effects of telerehabilitation on pain, physical function, and intermuscular adipose tissue concentration in patients with knee Osteoarthritis: a randomized controlled trial https://ensaiosclinicos.gov.br/rg/RBR-662hn2 [registered on 27-07-2019] Universal Trial Number U1111-1230-9517
Protocol version {3}	Version 1 of 27-07-2019; Version 2 (recruitment date updated - under review)
Funding {4}	This research is funded by the Coordenação de Aperfeiçoamento de Pessoal de Nível Superior - Brasil (CAPES) - Finance Code 001.
Author details {5a}	J.B.A.: Department of Physical Therapy, Federal University of São Carlos (UFSCar), Brazil. A.C.A.: Department of Physical Therapy, Federal University of São Carlos (UFSCar), Brazil. M.N.: Rural Health School, Community and Allied Health Department, La Trobe University, Australia. S.M.M.: Department of Physical Therapy, Federal University of São Carlos (UFSCar), Brazil.
Name and contact information for the trial sponsor {5b}	Investigator initiated clinical trial; S.M. Mattiello (Principal Investigator) stela@ufscar.br
Role of sponsor {5c}	This is an investigator initiated clinical trial. Therefore, the funder played no role in the design of the study; collection, analysis and interpretation of data; and in the decision to write and submit the manuscript for publication.

## Introduction

### Background and rationale {6a}

The knee joint is one of the most affected, leading to several functional, social, and economic consequences [1].

Obesity is one of the risk factors for OA. Muscle tissue is widely affected by fat accumulation, undergoing functional changes due to changes in the orientation of muscle fibers, thus decreasing the capacity to produce strength and physical function [1, 2]. A recent systematic review found that knee OA appears to lead to a higher amount of infiltrated fat in the thigh muscles when compared to people without knee OA regardless the body mass index (BMI) [3].

Among the therapeutic programs for patients with knee OA, physical exercise has shown to be promising in relieving pain and improving physical function [4]. Exercise protocols used to treat knee OA patients focus on improving aerobic capacity, quadriceps strength, and lower limb performance [5]. Therefore, a combination of both resistance and aerobic training has shown promising results for this population [6].

Circuit training (CRT) consists of a repetitive sequence of calisthenic exercises with shorter rest periods, which keeps the heart rate elevated throughout the training session [7]. This exercise model allows gain in muscle mass, improved body composition and functional capacity, and gain in muscle strength and cardiovascular benefits [8, 9].

Considering the benefits of CRT, our research group has been investigating the effects of a periodized circuit training protocol on non-obese individuals with knee OA, in different parameters, including inter and intramuscular fat [7]. Thus, a recent randomized controlled trial was conducted with this population, subjected to three different protocols: circuit training (CRT), strength training, and educational protocol [10]. The results showed that, in addition to the benefits of decreasing self-reported symptoms and physical function in the exercised groups, only the CRT group had a greater improvement in body composition measures, especially in thigh intermuscular adipose tissue. Thus, given that this exercise modality can bring significant benefits to individuals with knee OA, the disclosure of this therapy and patient adherence need to be tested.

Although the benefits of exercise are widely described, most people with knee OA do not meet the physical activity guidelines for good health [11], which are specific physical activity levels, such as frequency, intensity, and duration necessary for this purpose [12, 13]. Therefore, promoting continuous adherence is fundamental and a challenge for all involved professionals [14]. Physical therapists are often responsible for prescribing physical exercises for patients with knee OA. Physical therapy sessions are traditionally a face-to-face consultation, but for many people access to physical therapy is limited by physical distance, social isolation, and/or inability to afford the costs [15].

Telerehabilitation has been used adopting telecommunications technology as a service provider. Studies have shown that this treatment method provides improvements in physical activity and pain levels for people with chronic knee pain [16]. Additionally, face-to-face interventions, as well as those performed at distance, conducted electronically, resulted in similar clinical improvements after 12 weeks of treatment in patients with knee and hip OA [17]. Thus, telephone service models offer great potential to increase access to physical therapists and align with contemporary care models [18].

The implementation of any telerehabilitation method depends on the accessibility and familiarity of those receiving the intervention [19]. This means that the results of telerehabilitation can be influenced by geography (e.g., country) and socioeconomic status, both influencing the access and availability of the technologies needed for telerehabilitation. Countries such as the USA, Norway, Japan, and Australia are among the top 25 countries (out of 139 countries) in the Networked Readiness Index (NRI) [20]. Developing countries are ranked in the bottom half of the list of countries, with a considerable difference in NRI compared to the top countries [20]. To date, no study has evaluated the effectiveness of a telephone-type circuit training protocol in individuals with knee OA, specially conducted in a developing country.

Thus, it is essential that tools such as telerehabilitation be used to facilitate access, disseminate specific treatments, and increase adherence to physical exercise in this population. Therefore, comparing the effects of a face-to-face and telerehabilitation periodized circuit training protocol on the clinical characteristics of patients with knee OA seems relevant to clinical practice, as it may produce applicable results that are easily accessible to the health service. In addition, since the concentration of adipose tissue in the muscle has been described as a risk factor for the development and progression of knee OA, it is important to investigate the muscle morphological changes of patients with knee OA and its resulting clinical alterations.

## Objectives {7}

The primary aim of this randomized controlled trial is to determine if the periodized circuit training applied via telerehabilitation is just as effective to the same training applied face-to-face for improving pain intensity and physical function at 14 weeks (primary time-point) and 26 weeks (secondary time-point), in individuals with knee OA.

Secondary aims are to compare clinical (muscle strength and pain catastrophizing) and morphological characteristics (body composition, intermuscular adipose tissue, and muscle architecture), as well effectiveness, adherence, acceptability, and treatment perspectives 14 and 26 weeks after treatment. Safety endpoint will be determined by recording the number of adverse events during the intervention period.

## Trial design {8}

The present protocol is a non-inferiority randomized, parallel, single-blind trial with 1:1 allocation to the involved groups studied. This study was prospectively registered in the Brazilian Registry of Clinical Trials (ReBEC) under identifier RBR-662hn2 and with Universal Trial Number U1111-1230-9517. The following methodology is in full agreement with the OARSI (Osteoarthritis Research Society International) recommendations for randomized controlled trials (RCTs) [21], the Consolidated Standard of Reporting Trials (CONSORT) [22], and the SPIRIT (Standard Protocol Items for Randomized Trials) [23].

## Methods: participants, interventions, and outcomes

### Study setting {9}

Participants from the community of São Carlos, Brazil, will be invited to participate in this study. Participants will be recruited by ads in local newspapers, magazines, and social media. After declaring interest, participants will be phone interviewed by the Trial Coordinator to verify if they meet the primary eligibility criteria. If they were deemed eligible, a face-to-face evaluation will be scheduled to confirm the definitive eligibility criteria.

### Eligibility criteria {10}

#### Primary inclusion criteria (phone screening)

Participants must meet the following criteria to be eligible for the study:

i. Aged ≥ 40 years [24]
ii. Report knee joint pain
iii. Report history of knee pain ≥3 months

#### Primary exclusion criteria (phone screening)

Participants will be excluded during the phone screening if they:

i. Report body mass index (BMI) ≥ 30 kg/m^2^ [10, 25]
ii. Are currently consulting a physical therapist, walking more than 30 min continuously daily, or doing regular physical exercises (more than twice a week) up to 6 months prior to the beginning of this study [7]
iii. Have had previous knee surgery [7]
iv. Have used corticosteroid infiltration in the knees up to 6 months prior to this study [26]
v. Have a history of lower limb trauma within 30 days prior to evaluation [26]
vi. Have used chondroprotectors [26]
vii. Have rheumatic diseases [7]
viii. Have uncontrolled heart disease [26]
ix. Have history of severe muscle damage (above grade I) [7]
x. Have motor deficit due to neuromuscular disease [7]
xi. Have any medical restriction that precludes participation in this study [26]

Definitive eligibility will be screening during the first face-to-face evaluation based on the criteria below.

#### Definitive inclusion criteria (face-to-face evaluation)

Participants must meet the following criteria to be eligible for the study randomization:

i. Report an average pain score ≥ 400 mm on analog visual scale [10]
ii. Report at least one of the following: age > 50 years, morning knee stiffness ≤30 min, and crepitus [27]
iii. Radiographically diagnosed with unilateral or bilateral knee OA grade II or III according to the criteria of Kellgren and Lawrence (1957) [28]

For participants with bilateral OA, the most symptomatic side will be considered for evaluation.

#### Definitive exclusion criteria (face-to-face evaluation)

Participants will be excluded during the face-to-face evaluation if they:

i. Have cognitive impairment that compromise the understanding of the tests (obtained by Mini Mental State Examination (MMSE)) [29].

#### Screening definitive eligible criteria

##### Knee radiography

The presence of knee OA (K/L II and III) will be confirmed by radiographic examination. The exam will be performed bilaterally in the posteroanterior view with a 45° weight-bearing half-knee (orthostatic position), axial to the patellofemoral joint [30] and lateral, in the supine position at 45° of knee flexion. The images will be acquired and classified at the UFSCar University Hospital (UH) by an experienced radiologist.

##### Mini Mental State Examination (MMSE)

MMSE will be used as a screening test for cognitive loss, exclusion criteria of the present study. The MMSE consists of 11 items, divided into 2 sections. The first section requires verbal answers and questions for guidance, memory, and attention, and the second section is for reading and writing questions. The questions are performed in the order in which they are listed and can total a maximum score of 30. Thus, the following individuals will be considered to have cognitive impairment: illiterate with a total score of less than 20, individuals with 1 to 4 years of study with a total score less than 25, individuals with 5 to 8 years of study with a total score of less than 26.5, individuals with 9 to 11 years of study with a total score of less than 28, and individuals who studied for more than 11 years with a total score of less than 29 [31].

### Who will take informed consent? {26a}

All participants considered eligible by phone and face-to-face screening will receive oral and written information about the research procedures. The purpose, potential risks (e.g., muscular pain after doing exercise protocol and physical assessments), and possible benefits (e.g., improvement in daily activities and pain relief) involved in the study will be explained by the Trial Coordinator. After agreeing to participate, participants will sign an informed consent form. This study protocol has been approved by the Human Research Ethics Committee from the Federal University of São Carlos (CAAE: 05833118.6.0000.5504) and will be conducted according to the norms of National Health Council Resolution 466/2012 on research involving human beings.

### Additional consent provisions for collection and use of participant data and biological specimens {26b}

This randomized controlled trial will not use participant data and biological specimens from ancillary studies.

### Interventions

#### Explanation for the choice of comparators {6b}

According to the OARSI Clinical Trials Recommendations [21], usual care is a recommended comparator for interventions involving exercise. Thus, participants allocated to the control group will receive the face-to-face periodized circuit training protocol proposed by Almeida et al. [7] which has demonstrated improved physical function, muscle strength, and reduced intermuscular adipose tissue (IMAT) concentration in patients with knee OA [10].

#### Intervention description {11a}

##### Face-to-face (FtF) circuit protocol

Participants allocated to this group will attend all of their exercise sessions supervised by a trained physical therapist in the Federal University of São Carlos (UFSCar).

The exercise protocol will be performed in a group of up to 5 participants, three times a week, for 14 weeks, totaling 42 sessions [7]. Each session will consist of 5 min of warm-up (stationary bike or walk), circuit protocol, and 5 min of stretching exercises (cool down). Immediately after the warm-up, participants will begin the circuit protocol consisting of exercises for the upper limbs, lower limbs, trunk, and global exercises.

Participants will be instructed to perform each exercise as fast as possible, with the maximum number of repetitions during the time set for the training phase (light, moderate, or intense), in order to ensure the aerobic component [7]. After the first exercise, the participant should move to another station, following a specific order and allowing different muscle groups to alternate between rest and work, favoring recovery and minimizing the risk of muscle fatigue [7].

During the first week of exercise, participants will be familiarized with the equipment and exercises that will be performed during the protocol, performing stratified light exercises, where each exercise should be performed for only 10 s [7]. In the second, third and fifth weeks of training, participants will also perform exercises stratified as light, but each exercise will last 20 s. Moderate exercises will be performed in the sixth, eighth, and ninth weeks, lasting 30 s each. Intense exercises will be performed in the eleventh, twelfth, and fourteenth weeks, lasting 40 s each. The fourth, seventh, tenth, and thirteenth weeks will be considered as regenerative weeks, during which light exercises will be performed for a shorter time (10 s). For all intensities, a maximum transition interval of 30 s between stations will be implemented [7].

The exercises that compound each of the circuit training phases, as well as the execution time are presented in Additional file 1.

##### Telerehabilitation (TR) circuit protocol

Participants allocated to the TR group will be invited to attend the Department of Physical Therapy at UFSCar after randomization and before starting the intervention. This visit will be held individually so that the participant has full attention from the physical therapist to clarify questions, possible adaptations regarding the execution of exercises at home, as well as signs and symptoms to interrupt the session. In addition, during this visit, participants will receive a DVD with recordings of the circuit exercise sessions, the material needed to perform all the exercises, and a diary to record the days that they will perform the exercises. The materials provided to the participants are the same used in the FtF group and include a pair of 0.5-kg dumbbell, a pair of 1.0-kg dumbbell, a light elastic band, a hard-elastic band, a small ball, a 0.5-kg ankle weight, and a 1.0-kg ankle weight.

The TR group exercises will follow the same model proposed for the FtF group, following the same progression, execution time, and rest time (Additional file 1). However, these participants will perform the exercise sessions remotely.

Participants from this group will be instructed to perform the exercises scheduled for this week following the instructions given via DVD, website (http://exercicio-joelho.trekeducation.org), *YouTube* channel (http://bit.ly/telereabufscar), and/or videos via *WhatsApp*. The exercises should be performed three times weekly, preferably on non-consecutive days. Each session will consist of 5-min warm-up (light walking, steady walking), circuit exercises, and cool-down (5-min stretching), identical to the FtF group.

The TR group will also receive periodic phone calls from a single previously trained researcher/physical therapist. The phone calls will take place in weeks 2, 3, 4, 6, 8, 11, and 14; they will be recorded and will last approximately 20 min. In addition, it should be noted that phone calls will be made at participant’s preferred timed and will be motivating and instructive; however, participants will not be informed when calls will be performed. During these phone calls, a semi-structured interview will be performed, as proposed by Hinman et al. [32]. Table 1 presents the semi-structured interview guide. In addition, during these calls, it will be possible to detect possible difficulties with carrying out the exercises, as well as monitor the progress in subsequent calls, assessing the ability to perform the exercises, changes in symptoms, and functional capacity.

**Table 1:** Semi-structured interview guide

**Questions**	
1. How was your week?	
2. How are you feeling?	
3. Are you having difficulty performing the exercises?	
4. How often have you been doing the exercises and at what time?	
5. Is any family member doing the exercises with you?	
6. Is there something in your routine that you could modify to be able to perform the exercises?	
7. Do you think exercise is beneficial to your health?	

#### Criteria for discontinuing or modifying allocated interventions {11b}

Participants are free to withdraw from the study at any time and for any reason without penalty. The participant data that have been collected up to that moment will be included in the analysis.

#### Strategies to improve adherence to interventions {11c}

Adherence reminders will take place during the phone calls to the TR group, and during the face-to-face sessions to the FtF group. The researcher/physical therapist will provide support by increasing participants’ knowledge and understanding of knee OA, such as the causes of pain and the benefits of exercise. This will provide strategies for stimulating activity and dealing with pain during exercise, providing realistic expectations of likely outcomes, reaffirming that pain does not need to be feared and helping the participant to build confidence in their ability to perform exercises.

#### Relevant concomitant care permitted or prohibited during the trial {11d}

Permitted concomitant care comprises all drug prescriptions for medical conditions that do not meet the exclusion criteria of the present study. The prohibited care includes the uptake of new physical therapeutic measures.

### Provisions for post-trial care {30}

Trial center insurance will compensate for those who suffer harm from trial participation.

### Outcomes {12}

**Fig. 1:**
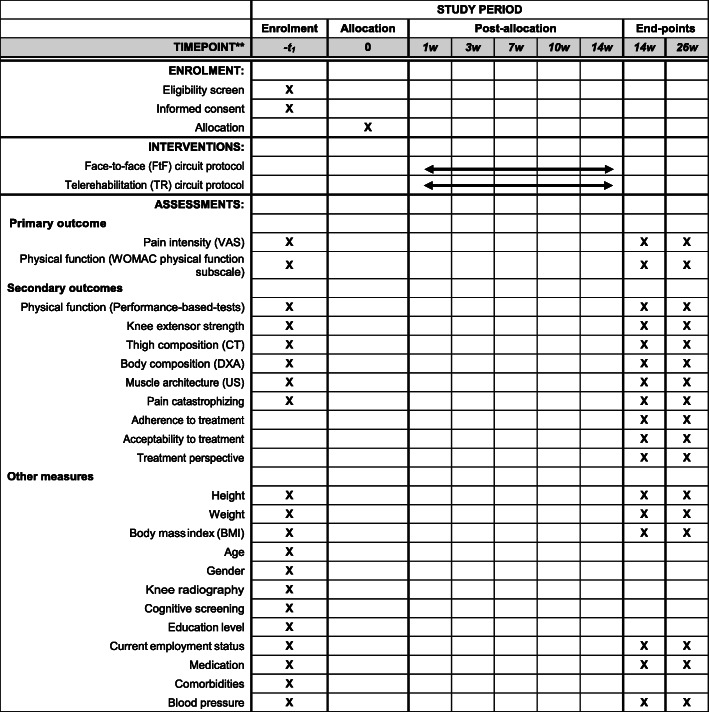
Schedule protocol. *Abbreviations*: FtF, face-to-face; TR, telerehabilitation; VAS, Visual Analog Scale; WOMAC, Western Ontario and McMaster Universities; CT, computed tomography; DXA, dual energy X-ray absorptiometry; US, ultrasound; BMI, body mass index

Figure 1 summarizes the schedule of enrolment, interventions and assessments for this study protocol according to SPIRIT recommendations [33].

#### Primary outcomes measurement

Pain and disability are the primary outcomes of this study protocol and the main symptoms that drive individuals with knee OA seek physical therapy care. In addition, assessing both of these symptoms are strongly recommended as outcomes of change in clinical trials with individuals with knee OA [34]. The primary end-point is 14 weeks and the secondary end-point is 26 weeks.

#### Pain Intensity

Pain intensity will be measured using Visual Analog Scale (VAS). This is a reliable measure of pain [35] consisting of a 100-mm line in which participants must mark between the left side (0, representing “no pain”) and 100 (representing “the worst pain imaginable”). This measure will be self-reported on a paper form.

#### Physical function subscale of the Western Ontario and McMaster universities osteoarthritis index (WOMAC)

To assess physical function, the Western Ontario and McMaster Universities (WOMAC) questionnaire will be used, a self-administered instrument that addresses the impact and restrictions on quality of life, specifically for patients with lower limb OA [36]. The questionnaire has been translated into Portuguese and validated [37], consisting of 24 questions divided into three domains: pain, stiffness, and physical function. The physical function subscale contains 17 questions, and the score is made using a Likert scale, whereby each question is scored between “no dysfunction” (score = 0) to “extreme dysfunction” (score = 4). Total score ranges from 0 to 68, with higher scores indicating worse function. Self-reported answers will be registered on a paper form.

#### Secondary outcomes measurement

All secondary outcomes will be also calculated using data measured at baseline, 14 weeks, and 26 weeks. Only adherence, acceptability, and treatment perspective will be measured at 14 weeks and 26 weeks.

#### Physical function—performance-based tests

The functional performance tests selected for this study follow the recommendations by OARSI (2013) [38]. All tests will be conducted by a single blinded evaluator on baseline and both end-points.

##### 40 m Fast-paced Walk Test

Participants will be instructed to walk as fast as possible without running for a distance of 10 m, returning to the starting point and repeating the walk for a total distance of 40 m. The test will be timed and the speed in meters/second (m/s) will be used for analysis [38, 39].

##### 30-second Chair Stand Test

From the sitting position, with feet flat on the floor and arms crossed over the chest, the participant should stand up completely from the chair and then sit down as quickly as possible for 30 s. The total number of repetitions (standing up and sitting represents one repetition) performed will be recorded [38, 40].

##### Stair climb test

The test will consist of ascending and descending a flight of 12 steps 16 cm high each. Participants should start the test standing on a line and, at the command to start, must climb to the top of the steps and immediately turn and descend as quickly as possible safely. The total test duration will be timed (in seconds); longer times indicate more impaired physical function [38, 40].

#### Knee extensors strength

The maximal isometric torque peak of the knee extensors will be measured using an isokinetic dynamometer (Multi-Joint System 3, Biodex Medical System, New York, USA) with a sampling frequency of 100 Hz by a single blinded evaluator. Knee extensors have been chosen as quadriceps weakness has been widely associated with knee OA [41].

The participant will be positioned on the equipment chair, with the knees flexed at 90^o^ and stabilized by non-elastic bands on the thorax, waist, and thigh of the limb to be tested. The dynamometer’s rotation axis will be aligned with the lateral femoral epicondyle and the movable arm fixed 5 cm above the medial malleolus. In addition, participants will be instructed to keep their arms crossed in front of their chest during contractions. Before performing the test and after receiving instructions, the participant must perform 3 submaximal isometric contractions to become familiar with the test procedure. After familiarization, the participant will be instructed to rest for 2 min.

For isometric extensor torque evaluation, the knee will be positioned at the 60° of flexion angle. The test will consist of 3 maximum contractions held for 3 s each, with a 1-min rest between contractions. Standard verbal encouragement will be given during the test [41–43].

The isometric peak torque will be calculated as the average of the three contractions [44]. The isometric mean peak will be normalized by body mass (kg) (mean isometric peak torque/body mass × 100) and used for statistical analysis.

#### Thigh composition

The CT will be performed to assess thigh composition (intermuscular fat, subcutaneous fat, muscle mass and mean muscle attenuation). The images will be obtained through a Multislice Tomograph (Brilliance CT 16-slice, Phillips), located at UH-UFSCar, by an experienced radiologist.

For image acquisition, the criteria according to Eastwood et al. [45] will be followed, comprising the following parameters: helical mode, 120 KV, 150 mAs, slice thickness = 5 mm, and 50 cm field of view. The suggested protocol will provide a mid-thigh image of the most affected limb. To quantify the thigh tissues, the images will be obtained at the midpoint between the greater trochanter and the femoral intercondylar fossa. During the exam, the participant will remain in the supine position, with their hands resting on the head and arms flexed at 90° for approximately 3 min.

Thigh composition analyses will be performed manually using the ITK-SNAP software (version 3.6) [10] by a single evaluator blinded to the interventions group. The 2 middle slices will be selected for segmentation, representing 10 mm of the area of interest. Thus, the area of interest of each slice will be selected by scanning according to the attenuation rates for quantification of adipose tissue present in cm^2^. In addition, a manual line will also be performed separating the bony part from the soft tissues and then the adipose tissue present within the bone area will be subtracted from the area obtained by measuring intermuscular and subcutaneous adipose tissue.

Skeletal muscle and adipose tissue areas will be calculated by the attenuation value range for skeletal muscle tissue (0 to 100 Hounsfield) and adipose tissue (−190 to −30 Hounsfield) [46, 47]. The mean muscle attenuation will be calculated by the average of the Hounsfield values obtained in the muscle fascia area. The intra-rater reliability performed at 1-week interval was ICC = 0.97 and interrater reliability was ICC = 0.99 (*n* = 20).

#### Body composition

For body composition analysis, the reference standard will be the Dual Energy X-ray absorptiometry (DXA) device (Discovery A, Hologic), which uses the three-compartment model (lean body mass, adipose tissue, and bone mineral density). This technique estimates the body composition in the whole and by body segment. Scanning will be performed according to the manufacturer’s recommendations. Thus, the operator should check that no metal objects remain in the scanning area [48]. Participants will be asked to attend in a fasted state (at least 4 h) and not to perform any physical activity within 24 h prior to the exam [7]. The participant will be placed in the supine position and should remain un-moving during the exam. The device software will automatically define areas of regional body estimates (left and right arms, legs and trunk) [48]. For this study, it was defined that the segment areas and total body composition will be used for analysis.

#### Muscle architecture

The pennation angle (PA), muscle thickness (MT), and fascicle length (FL) of the vastus lateralis muscle will be obtained using an ultrasound device (US) (Acuson X300 PE, Siemens) and linear transducer (4–11.4 MHz). The same previously trained evaluator, blinded to intervention groups, will obtain all images. The vastus lateralis was the chosen muscle for analysis due to the simple fascicle alignment when compared to the other quadriceps muscles [49].

For image acquisition, the participants will be positioned supine with legs extended and relaxed muscles [50]. Three vastus lateralis images will be collected at the midpoint of the thigh, measured as the midpoint between the greater trochanter and the lateral femoral epicondyle [49]. Water-soluble gel will be applied between the transducer and the skin to aid in acoustical coupling and to prevent pressure muscle deformation. The transducer will be oriented parallel to the muscle fascicles during the image acquisition.

A second investigator, also blinded to interventions group, will manually review all obtained images using the ImageJ software (National Institutes of Health, USA). PA will be defined as the insertion angle of the fascicle in the deep aponeurosis [51]. MT will be defined as the distance between deep and superficial aponeurosis [44, 49]. FL will be defined as the distance between the origin of the fascicle in the superficial aponeurosis and the insertion of the same fascicle in the deep aponeurosis [51–53]. The average of the three MT, PA, and FL measurements will be used for analyses.

The intra-rater reliability performed at 1-week interval was ICC = 0.97 (*n* = 10).

#### Pain catastrophizing

The Pain Catastrophizing Scale (PCS) is a valid, reliable, and self-applicable 13-item scale that describes thoughts and feelings that people may experience when they experience pain [54]. Items are evaluated using a 5-point Likert scale, ranging from 0 to 4. The total score is calculated by the sum of the scores for all items (total score, 0–52). Higher scores indicate greater catastrophic thoughts about pain. A score greater than 30 represents a clinically relevant level of catastrophism [54].

#### Adherence, acceptability, and treatment perspective

Adherence/acceptability to TR, as proposed by Hinman et al. [32] will be assessed by the number of calls received from the lead investigator, as well as the number of sessions per week the participant performed, which should be self-reported during phone calls. The FtF will be evaluated by the number of sessions that the participant attended. In addition, for the same purpose, questions will be asked such as “Do you agree with the proposed exercise plan?” and “Did you perform the exercise program?” Where 0 refers to “strongly disagree” and 10 “strongly agree”. The participants will also qualitatively evaluate the expectation with the result of the exercise protocol, where the scale may vary from “no effect” to “complete recovery” [32]. These outcomes will be self-reported on a paper form only during end-points (14 weeks and 26 weeks).

### Participant timeline {13}

**Fig. 2:**
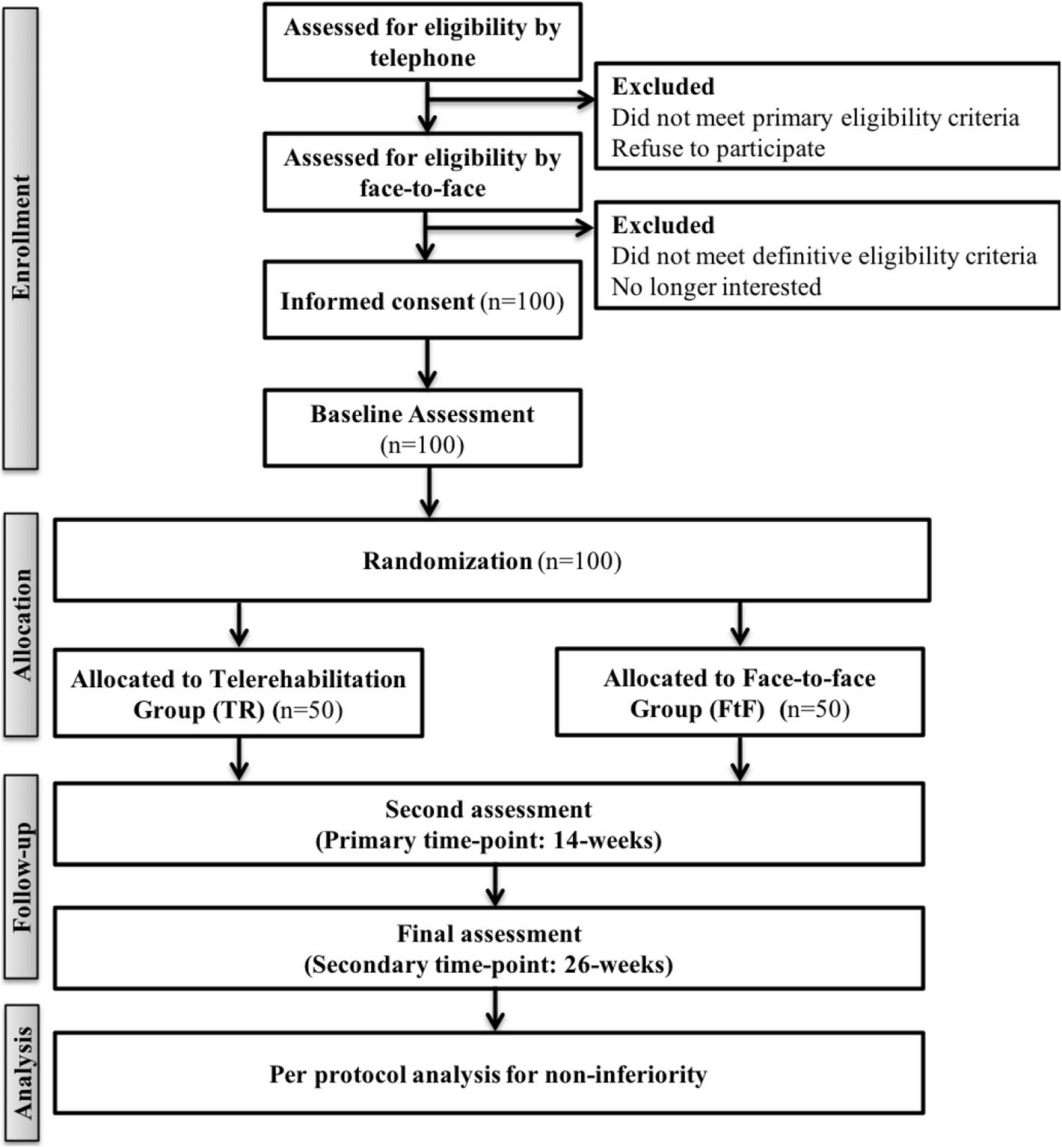
Flow diagram showing the steps participants take in the RCT

The flow of participants through the present RCT is presented in Fig. 2.

### Sample size {14}

Sample size was based on detecting non-inferiority of telerehabilitation circuit training in comparison to the face-to-face method at 14 weeks after randomization, considering the primary outcomes pain (VAS) and disability (WOMAC function sub-scale). For change in VAS, a non-inferiority margin (NIM) of 16 mm was chosen as this is less than the minimum clinically important difference (MCID) of 17.5 mm from a 100-mm VAS for OA patients [55]. For change in WOMAC function sub-scale, a NIM of 7.5 units (11% of scored 0–68) was chosen as this is less than the MCID of 12% (8.16) of improvement from baseline in OA research [56]. Assuming standard deviations (SD) for changes from baseline of 17.9 mm and 12.12 units for pain intensity and physical function respectively [10, 25], 80% power and a one-sided 2.5% significance level, we needed 20 people per arm for change in pain intensity and 41 people per arm for change in physical function. However, considering 18% loss to follow-up, we needed 25 and 50 people per arm for change in pain intensity and physical function, respectively. Thus, a total of 100 people will be included in the trial. No adjustments for multiplicity was made. Sample size calculation was performed using the software G*Power, version 3.1.

### Recruitment {15}

Participants from the community of São Carlos, Brazil, will be recruited by ads in local newspapers, magazines and social media.

## Assignment of interventions: allocation

### Sequence generation {16a}

Participants will be randomly assigned to the intervention group through the stratified randomization method. This method can be used to achieve balance among groups in terms of participants’ baseline characteristics. Thus, participants will be paired into two groups (allocation 1:1) considering similar baseline characteristics, such as sex, age, BMI, and knee OA grade. Once paired, a simple randomization will be performed through a randomization site (http://www.random.org/) to define the intervention method of each group (TR and FtF).

### Concealment mechanism {16b}

A single investigator, who will have access only to participants’ baseline characteristics, such as sex, age, BMI, and knee OA grade, will be responsible to pair participants as explained under sequence generation section. Then, the same investigator will perform a simple randomization through a website which will decide the intervention received by each participant (TR of FtF). The investigator will inform both the participants and physical therapist responsible to interventions.

### Implementation {16c}

All patients who give consent for participation and who fulfill the inclusion criteria will be randomized. Randomization will be requested by the Trial Coordinator after conclusion of the baseline assessments and after pairing participants (1:1). The estimated time between baseline assessments and allocation will not exceed 7 days. To make RCT feasible, the study sample will be divided into three stages: the first stage will recruit 20 participants (10 per arm), and the second and third stages will recruit 40 participants each (20 per arm), totaling 100 participants.

## Assignment of interventions: blinding

### Who will be blinded {17a}

The outcome assessors and the data analysts will be blinded to the treatment groups. Due to the nature of the interventions, it will not be possible to blind neither the patients nor the physical therapist responsible to interventions. Thus, it is important to note that the primary outcomes (pain intensity, VAS; and disability, WOMAC function sub-scale), as well as pain catastrophizing, adherence, acceptability and treatment perspective are patient reported, and hence they cannot be blinded.

### Procedure for unblinding {17b}

The trial design is a single-blinded study; thus, only the outcome assessors and the data analysts will be blinded. Thus, there is no unblinding procedure foreseen.

## Data collection and management

### Plans for assessment and collection of outcomes {18a}

Assessments will be conducted face-to-face at the Joint Function Analysis Laboratory (LAFAr) and at the University Hospital (UH) of the UFSCar and will be divided into 3 days.

At the first assessment day, participants will undergo a physical therapy evaluation consisting of inspection, palpation, and knee and hip range of movement analysis, as well as tests to assess ligament integrity. Afterwards, an evaluation form will be completed (personal, anthropometric, socioeconomic, medication, blood pressure, knee pain data, and previous medical history). In addition, the body composition exam (dual energy X-ray absorptiometry (DXA)) and the performance-based tests will be held in the same visit. This first day of assessment will be performed by the same physical therapist/researcher and will have a total duration time of approximately one hour.

The second assessment day will be scheduled at least 2 days after the first assessment. In this visit, the WOMAC questionnaire, the Pain Catastrophizing Scale (PCS) and the Visual Analog Scale (VAS) will be participant-reported on a paper form. Then, participants will undergo an ultrasound evaluation of the thigh vastus lateralis muscle by a previously trained evaluator, blinded to intervention groups. Afterwards, participants will perform the isometric strength test. The second assessment day will also be conducted by the same physical therapist/researcher and will have a total duration time of approximately one hour.

Finally, participants will receive a referral for the thigh computed tomography (CT) scan exam that composes the third assessment day. The CT will be performed at the University Hospital (UH) – UFSCar by an experienced radiologist collaborator of this study. The CT exam has a duration time of approximately 10 min. More detailed information on the data collection for these study outcomes was provided in the “Outcomes {12}” section.

Adherence, acceptability, and treatment perspective will be self-evaluated during the end-points (14 weeks and 26 weeks). Thus, questions related to these outcomes will be included in the first day of assessment post-intervention.

The assessments will be conducted at all timepoints (baseline, 14 weeks, and 26 weeks) by the same physical therapist/researcher. It is important to note that the CT exam will also be performed by the same radiologist. Both physical therapist/researcher and radiologist will be blinded to the intervention’s groups. In addition, all randomized participants will be invited to attend all timepoints of the study, even if they do not adhere to the interventions.

### Plans to promote participant retention and complete follow-up {18b}

Participants will receive extensive information about the study design and requirements during the recruitment. The importance of the follow-up completion will be informed. Participants are allowed to withdraw from the study at any time and for any reason without penalty. However, if possible, participants will be invited to attend the end-point assessment of the present study (14 weeks and 26 weeks).

### Data management {19}

After checking for completeness, the data will be entered in an electronic password-accessible database by a blinded collaborator of this study. In a separate step, the correct transfer and coding of the data will be checked by a different blinded collaborator. All research data will be archived for 10 years after the study has ended according to the local ethics committee.

### Confidentiality {27}

The present study data will be documented and archived using an identification code for each participant. All personal information will be stored in a locked, password-accessible database. Paper forms and exams report containing personal information will be locked in a lockable metal cabinet. Physical and electronic personal documents will be safeguarded by the Trial Coordinator for 10 years as recommended by the local ethics committee; after that, they will be destroyed and deleted.

### Plans for collection, laboratory evaluation, and storage of biological specimens for genetic or molecular analysis in this trial/future use {33}

No biological specimens will be collected.

## Statistical methods

### Statistical methods for primary and secondary outcomes {20a}

A blinded data analyst will perform all analysis using the Statistical Package for Social Science, version 20.0 (SPSS Inc, Chicago, IL, USA). Baseline characteristics of participants will be presented using descriptive statistics. The between-group differences and 95% confidence interval (CI) for the post-treatment outcomes at 14-week and 26-week end-points will be calculated using mixed linear models using interaction terms of treatment group versus time with no covariate adjustment. Non-inferiority will be demonstrated if the lower bound of the two-sides 95% CI for between-group difference (telerehabilitation minus face-to-face) is above −16 mm in pain intensity and/ or −7.5 units for change in WOMAC physical function subscale at 14 weeks after randomization. Multiple imputation method will be used to impute missing data by using the multiple imputations function in SPSS [57], and an intention-to-treat analysis [58] (including all randomized participants) will be conducted. No adjustments for multiplicity will be made.

It is important to note that, if appropriate, a full statistical plan will be finalized prior to analysis.

### Interim analyses {21b}

There are no interim analyses planned.

### Methods for additional analyses (e.g., subgroup analyses) {20b}

There are no subgroup analyses planned.

### Methods in analysis to handle protocol non-adherence and any statistical methods to handle missing data {20c}

The primary and secondary outcomes will be assessed using an intention-to-treat-analysis. Multiple imputation method will be used to impute missing data.

### Plans to give access to the full protocol, participant-level data, and statistical code {31c}

The full protocol, participant-level data, and statistical code can be made available by the Trial Coordinator upon reasonable request.

## Oversight and monitoring

### Composition of the coordinating center and trial steering committee {5d}

The coordinating center is located at the Department of Physical Therapy at the Federal University of São Carlos and comprises the Trial Coordinator, the principal investigator, the radiologist doctor, the radiologist technician, 5 physical therapists, 2 computer scientists, and the data analysts. The study team meets weekly. There is no trial steering committee or public involvement group.

### Composition of the data monitoring committee, its role, and reporting structure {21a}

A data monitoring committee was not considered as this study adopts a low-risk intervention, and an interim analysis is not planned in this study.

### Adverse event reporting and harms {22}

Participants who experience any adverse event are instructed to report these to their physical therapist. All adverse events reported by the participant or observed by the physical therapist during sessions will be recorded. In this RCT, adverse events are defined as any problem experienced during the execution of the study as a result of the assessments, exercise protocol, and/or advice given by the physical therapist.

### Frequency and plans for auditing trial conduct {23}

The researchers group meets every week to discuss the trial development and upcoming issues. The study documents, such as informed consents, inclusion and exclusion criteria, and source data are checked four times a year for an independent researcher, who is not involved with the present study. If a document is found to be missing or information is inconsistent, the local ethics committee is notified immediately.

### Plans for communicating important protocol amendments to relevant parties (e.g., trial participants, ethical committees) {25}

All amendments to the protocol will be communicated and approved by the Human Research Ethics Committee from the Federal University of São Carlos-SP.

### Dissemination plans {31a}

The results of this RCT will be disclosed completely in international peer-reviewed journals. Both positive and negative results will be reported. In addition, participants will receive a report with all the results of the study.

## Discussion

Despite the fact that effects of physical exercise on the knee OA population are already widely investigated, the periodized circuit training model is still innovative for treating symptoms and functionality for this population and has resulted in promising findings. In addition, the focus on body composition, especially localized measures such as intermuscular adipose tissue, considering all its associated dysfunctions, such as impairment of muscle architecture and consequent reduction of muscle strength, should be considered in clinical practice, especially when prescribing physical training.

Furthermore, as previously mentioned, there are important barriers in adhering to physical therapy treatment by many patients due to logistical and financial difficulties, especially in developing countries. No studies were found which investigated the effects of remote training, such as telerehabilitation, using a periodized circuit training protocol, compared to face-to-face training in this population, with a randomized controlled trial model.

As previously mentioned, circuit training promotes changes in body composition and muscle strengthening, as well as being a training modality tolerated by patients with knee OA, as attested in our previous studies. The benefits of this modality practiced remotely should be better explored considering its diverse benefits in both skeletal muscle and cardiorespiratory systems. In addition to the physical benefits, it is worth emphasizing the financial benefits that this modality provides, considering the possibility of remote treatments, but still with guidance from the physical therapist.

Given the lack of information in the published literature and the public health impact that exercise intervention protocols can provide, the effects of telerehabilitation using a periodized circuit training protocol on thigh composition (adipose tissue and muscle mass), pain, physical function, muscle architecture, and muscle strength are critical to delineate. Thus, through the study, we hope to provide clinicians with high-quality evidence of treatment for patients with knee OA, providing an effective way to deliver treatment for those who do not have access to face-to-face physical therapy treatment.

This study follows the OARSI recommendations for designing clinical trials for patients with knee OA [21], as well as the SPIRIT recommendations for performing randomized controlled trials [23]. Thus, considering the high prevalence and several disorders related to knee OA, as well as the gaps in the scientific literature regarding telerehabilitation, assessing the efficacy of a periodized circuit training protocol with a design focused on remote rehabilitation, and its impact on body composition, as well as on the several related outcomes, has immediate and high clinical impacts. The results of this study will provide critically needed guidance to the health care system for the treatment and prevention of complications related to knee OA.

Limitations of the study include the fact that before starting the intervention period the participants of the telerehabilitation group will be invited to attend a face-to-face session to clarify questions related to remotely exercises and to provide necessary materials. In addition, exercise participation will be self-reported (as opposed to be measured via accelerometers and similar); therefore, we will not know whether what is reported matches actual participation for the telerehabilitation group.

## Trial status

The protocol was registered on 31 March 2019, under the registration RBR-662hn2. The first two blocks of recruitment, allocation and analysis have already been completed. However, the final block of participants (*n* = 40) did not start yet due to the delay caused by the COVID-19 pandemic. Data recruitment began: 01 April 2019. Data when recruitment will be completed: 30 July 2021 - Version 2 (recruitment date updated - under review).

## Supplementary Information

**Additional file 1:.** Circuit Training Protocol Exercises

## Abbreviations

OA: Osteoarthritis
BMI: Body mass index
TR: Telerehabilitation
FtF: Face-to-face
VAS: Visual analog scale
PCS: Pain Catastrophizing Scale
IMAT: Intermuscular adipose tissue
CRT: Circuit training
NRI: Networked Readiness Index
OARSI: Osteoarthritis Research Society International
RCT: Randomized controlled trial
CONSORT: Consolidated Standard of Reporting Trials
SPIRIT: Standard Protocol Items for Randomized Trials
K/L: Kellgren and Lawrence
MMSE: Mini Mental State Examination
WOMAC: Western Ontario and McMaster Universities
UFSCar: Federal University of São Carlos
LAFAr: Joint Function Analysis Laboratory
UH: University Hospital
DXA: Dual Energy X-ray absorptiometry
CT: Computed tomography
ICC: Intraclass correlation
PA: Pennation angle
MT: Muscle thickness
FL: Fascicle length
US: Ultrasound
NIM: Non-inferiority margin
MCID: Minimum clinically important difference
SPSS: Statistical Package for Social Science
