# Supplementary material for: Effects of a periodized circuit training protocol delivered by telerehabilitation compared to face-to-face method for knee osteoarthritis: a protocol for a non-inferiority randomized controlled trial
Source: Trials. 2021 Dec 6;22:887. doi: 10.1186/s13063-021-05856-8 (PMC8646353; doi:10.1186/s13063-021-05856-8)
Supplement: Supplementary file 1 — Additional file 1:. Circuit Training Protocol Exercises [file 13063_2021_5856_MOESM1_ESM.docx]

**Additional file 1 -** Circuit Training Protocol Exercises

| **Intensity** | **Exercise time** | **Week (s)** | **Exercise** | **Position** | **Weight** | **Session duration** |
| --- | --- | --- | --- | --- | --- | --- |
| Light | 10 seconds | 1, 4, 7, 10, 13 | Shoulder adduction with elbow flexion | Supine | 0.5kg dumbbell | 15 minutes |
|  |  |  | Knee flexion - Right leg | Sitting | Light elastic band |  |
|  |  |  | Knee plank (isometric) | Ventral | No |  |
|  |  |  | Elbow flexion associated with contralateral hip and knee flexion | Standing | 0.5kg dumbbell |  |
|  |  |  | Alternating punches | Standing | No |  |
|  |  |  | Knee flexion - Left leg | Sitting | Light elastic band |  |
|  |  |  | Knee side plank (isometric) - Right side | Side lying | No |  |
|  |  |  | Shoulders abduction associated with hip flexion | Standing | 0.5kg dumbbell |  |
|  |  |  | Elbow flexion | Standing | 0.5kg dumbbell |  |
|  |  |  | Step-up and down | Standing | No |  |
|  |  |  | Knee side plank (isometric) - Left side | Side lying | No |  |
|  |  |  | Adapted Jumping Jack (no jumping) | Standing | No |  |
|  |  |  | Shoulder adduction with elbow flexion | Supine | 0.5kg dumbbell |  |
|  |  |  | Hip abduction - Right leg | Standing | 0.5 ankle weight |  |
|  |  |  | Abdominal curl with legs leaning on a chair | Supine | No |  |
|  |  |  | Elbow flexion associated with contralateral hip and knee flexion | Standing | 0.5kg dumbbell |  |
|  |  |  | Alternating punches | Standing | No |  |
|  |  |  | Hip abduction - Left leg | Standing | 0.5 ankle weight |  |
|  |  |  | Knee plank (isometric) | Ventral | No |  |
|  |  |  | Shoulders abduction associated with hip flexion | Standing | 0.5kg dumbbell |  |
|  |  |  | Elbow flexion | Standing | 0.5kg dumbbell |  |
|  |  |  | Calf strengthening | Standing | No |  |
|  |  |  | Abdominal curl with legs leaning on a chair | Supine | No |  |
|  |  |  | Adapted Jumping Jack (no jumping) | Standing | No |  |

**Additional file 1 -** Circuit Training Protocol Exercises **(continued)**

| **Intensity** | **Exercise time** | **Week (s)** | **Exercise** | **Position** | **Weight** | **Session duration** |
| --- | --- | --- | --- | --- | --- | --- |
| Light | 20 seconds | 2 | Shoulder adduction with elbow flexion | Supine | 0.5kg dumbbell | 15 minutes |
|  |  |  | Knee flexion - Right leg | Sitting | Light elastic band |  |
|  |  |  | Knee plank (isometric) | Ventral | No |  |
|  |  |  | Elbow flexion associated with contralateral hip and knee flexion | Standing | 0.5kg dumbbell |  |
|  |  |  | Alternating punches | Standing | No |  |
|  |  |  | Knee flexion - Left leg | Sitting | Light elastic band |  |
|  |  |  | Knee side plank (isometric) - Right side | Side lying | No |  |
|  |  |  | Shoulders abduction associated with hip flexion | Standing | 0.5kg dumbbell |  |
|  |  |  | Elbow flexion | Standing | 0.5kg dumbbell |  |
|  |  |  | Step-up and down | Standing | No |  |
|  |  |  | Knee side plank (isometric) - Left side | Side lying | No |  |
|  |  |  | Adapted Jumping Jack (no jumping) | Standing | No |  |
|  |  |  | Shoulder adduction with elbow flexion | Supine | 0.5kg dumbbell |  |
|  |  |  | Hip abduction - Right leg | Standing | 0.5 ankle weight |  |
|  |  |  | Abdominal curl with legs leaning on a chair | Supine | No |  |
|  |  |  | Elbow flexion associated with contralateral hip and knee flexion | Standing | 0.5kg dumbbell |  |
|  |  |  | Alternating punches | Standing | No |  |
|  |  |  | Hip abduction - Left leg | Standing | 0.5 ankle weight |  |
|  |  |  | Knee plank (isometric) | Ventral | No |  |
|  |  | 3 | Week 2 exercises |  |  | 20 minutes |
|  |  |  | Shoulders abduction associated with hip flexion | Standing | 0.5kg dumbbell |  |
|  |  |  | Elbow flexion | Standing | 0.5kg dumbbell |  |
|  |  |  | Calf Strengthening | Standing | No |  |
|  |  |  | Abdominal curl with legs leaning on a chair | Supine | No |  |
|  |  |  | Adapted Jumping Jack (no jumping) | Standing | No |  |
|  |  |  | Shoulder adduction with elbow flexion | Supine | 0.5kg dumbbell |  |

**Additional file 1 -** Circuit Training Protocol Exercises **(continued)**

| **Intensity** | **Exercise time** | **Week (s)** | **Exercise** | **Position** | **Weight** | **Session duration** |
| --- | --- | --- | --- | --- | --- | --- |
| Light | 20 seconds | 5 | Week 3 exercises |  |  | 25 minutes |
|  |  |  | Hip flexion with single leg raises - Right leg | Supine | 0.5 ankle weight |  |
|  |  |  | Knee plank (isometric) | Ventral | No |  |
|  |  |  | Elbow flexion associated with contralateral hip and knee flexion | Standing | 0.5kg dumbbell |  |
|  |  |  | Alternating punches | Standing | No |  |
|  |  |  | Hip flexion with single leg raises - Left leg | Supine | 0.5 ankle weight |  |
|  |  |  | Knee side plank (isometric) - Right side | Side lying | No |  |
| Moderate | 30 seconds | 6 | Alternating punches | Standing | 0.5kg dumbbell | 20 minutes |
|  |  |  | Hip abduction - Right leg | Standing | Light elastic band |  |
|  |  |  | Abdominal curl up holding a ball | Supine | Small ball |  |
|  |  |  | Elbow flexion associated with contralateral hip and knee flexion | Standing | 1.0kg dumbbell |  |
|  |  |  | Elbow flexion | Standing | 0.5kg dumbbell |  |
|  |  |  | Hip abduction - Left leg | Standing | Light elastic band |  |
|  |  |  | Bridge exercise with hip adduction holding a ball between knees | Supine | Small ball |  |
|  |  |  | Shoulders abduction associated with hip flexion | Standing | 1.0kg dumbbell |  |
|  |  |  | Shoulder flexion | Standing | Light elastic band |  |
|  |  |  | Hip adduction - Right leg | Standing | Light elastic band |  |
|  |  |  | Plank (isometric) | Supine | No |  |
|  |  |  | Adapted Jumping Jack (no jumping) | Standing | 0.5kg dumbbell |  |
|  |  |  | Elbow flexion | Standing | Light elastic band |  |
|  |  |  | Hip adduction - Left leg | Standing | Light elastic band |  |
|  |  |  | Side plank - Right side | Side lying | No |  |
|  |  |  | Squat holding a ball | Standing | Small ball |  |
|  |  |  | Alternating punches | Standing | 0.5kg dumbbell |  |
|  |  |  | Sit-to-stand |  | No |  |

**Additional file 1 -** Circuit Training Protocol Exercises **(continued)**

| **Intensity** | **Exercise time** | **Week (s)** | **Exercise** | **Position** | **Weight** | **Session duration** |
| --- | --- | --- | --- | --- | --- | --- |
| Moderate | 30 seconds | 6 | Side plank - Left side | Side lying | No |  |
|  |  |  | Elbow flexion associated with contralateral hip and knee flexion | Standing | 1.0kg dumbbell |  |
|  |  | 8 | Week 6 exercises |  |  | 25 minutes |
|  |  |  | Elbow flexion | Standing | 0.5kg dumbbell |  |
|  |  |  | Knee extension - Right side | Sitting | 0.5 ankle weight |  |
|  |  |  | Abdominal curl holding a ball | Supine | Small ball |  |
|  |  |  | Shoulders abduction associated with hip flexion | Standing | 1.0kg dumbbell |  |
|  |  |  | Knee extension - Left side | Sitting | 0.5 ankle weight |  |
|  |  | 9 | Week 8 exercises |  |  | 30 minutes |
|  |  |  | Shoulder flexion | Standing | Light elastic band |  |
|  |  |  | Bridge exercise with hip adduction holding a ball between knees | Supine | Small ball |  |
|  |  |  | Adapted Jumping Jack (no jumping) | Standing | 0.5kg dumbbell |  |
|  |  |  | Elbow flexion | Standing | Light elastic band |  |
| Intense | 40 seconds | 11 | Alternating punches | Standing | 1.0kg dumbbell | 25 minutes |
|  |  |  | Hip abduction - Right leg | Standing | Hard elastic band |  |
|  |  |  | Single leg bridge static hold - Right leg | Supine | No |  |
|  |  |  | Adapted Jumping Jack (no jumping) | Standing | 1.0kg dumbbell |  |
|  |  |  | Shoulder flexion | Standing | Hard elastic band |  |
|  |  |  | Hip abduction - Left leg | Standing | Hard elastic band |  |
|  |  |  | Single leg bridge static hold - Left leg | Supine | No |  |
|  |  |  | Criss cross | Standing | 1.0kg dumbbell |  |
|  |  |  | Elbow flexion | Standing | Hard elastic band |  |
|  |  |  | Hip adduction - Right leg | Standing | Light elastic band |  |
|  |  |  | Oblique abdominal strengthening holding a ball | Supine | Small ball |  |
|  |  |  | Squat plus overhead press | Standing | 0.5kg dumbbell |  |

**Additional file 1 -** Circuit Training Protocol Exercises **(continued)**

| **Intensity** | **Exercise time** | **Week (s)** | **Exercise** | **Position** | **Weight** | **Session duration** |
| --- | --- | --- | --- | --- | --- | --- |
| Intense | 40 seconds | 11 | Alternating punches | Standing | 1.0kg dumbbell | 25 minutes |
|  |  |  | Hip adduction - Left leg | Standing | Light elastic band |  |
|  |  |  | Bridge on pillow | Supine | No |  |
|  |  |  | Adapted Jumping Jack (no jumping) | Standing | 1.0kg dumbbell |  |
|  |  |  | Shoulder flexion | Standing | Hard elastic band |  |
|  |  |  | Knee flexion - Right leg | Standing | 1.0 ankle weight |  |
|  |  |  | Abdominal curl with hip flexion and knee extension holding a ball | Supine | Small ball |  |
|  |  |  | Criss cross | Standing | 1.0kg dumbbell |  |
|  |  |  | Knee flexion - Left leg | Standing | 1.0 ankle weight |  |
|  |  | 12 | Week 11 exercises |  |  | 30 minutes |
|  |  |  | Elbow flexion | Standing | Hard elastic band |  |
|  |  |  | Oblique abdominal strengthening holding a ball | Supine | Small ball |  |
|  |  |  | Squat plus overhead press | Standing | 0.5kg dumbbell |  |
|  |  |  | Alternating punches | Standing | 1.0kg dumbbell |  |
|  |  | 14 | Week 12 |  |  | 35 minutes |
|  |  |  | Hip abduction - Right leg | Standing | Hard elastic band |  |
|  |  |  | Single leg bridge static hold - Right leg | Supine | No |  |
|  |  |  | Adapted Jumping Jack (no jumping) | Standing | 1.0kg dumbbell |  |
|  |  |  | Hip abduction - Left leg | Standing | Hard elastic band |  |
|  |  |  | Single leg bridge static hold - Left leg | Supine | No |  |
